# Surgeons and social media: The use of twitter hashtags at the Academic Surgical Congress 2015–2019: A cross sectional study

**DOI:** 10.1016/j.amsu.2020.09.004

**Published:** 2020-09-06

**Authors:** Kristen Santarone, Evander Meneses, Aaron Shepherd, Dessy Boneva, Mark Mckenney, Adel Elkbuli

**Affiliations:** a Department of Surgery, Division of Trauma and Surgical Critical Care, Kendall Regional Medical Center, Miami, FL, USA; b Department of Surgery, University of South Florida, Tampa, FL, USA

**Keywords:** Academic surgical congress, Association for academic surgery, Healthcare hashtags, Knowledge dissemination, Social media engagement, Society of university surgeons, Twitter

## Abstract

**Background:**

The use of Twitter hashtags at medical conferences has revolutionized the way healthcare professionals interact and advance their education. We aim to investigate the scope of the Academic Surgical Congress's online reach and engagement through the use of Twitter hashtags #ASC from 2015 to 2019, by analyzing the number of impressions and tweets and retweets.

**Methods:**

A cross sectional study of Twitter data through Symplur with the following conference hashtags for the Academic Surgical Congress annual meetings for years 2015–2019: #ASC2015, #ASC2016, #ASC2017, #ASC2018, and #ASC2019. Data on tweets, retweets, users, and impressions was reviewed along with information on the top 10 influencers and the most frequently tweeted links. Symplur Signals software was utilized to extract and assimilate data. Statistical Significance was defined as p < 0.05.

**Results:**

Twitter engagement metrics significantly increased from 11,400 to 32,100 from 2015 to 2017 (p < 0.05). However, from 2017 to 2019, there was a significant decline in engagement metrics from 32,100 to 26,100 (p < 0.05). Impressions increased significantly from 13,100 in 2015 to 71,800 impressions in 2019 (p < 0.05). Users grew significantly from 1500 in 2015 to peak at 4600 in 2017 before dropping back to 3300 in 2019 (p < 0.05). The most influential organizations during these years were the organizers of the conference: Association for Academic Surgery and the Society of University Surgeons. Conference attendance progressively increased from approximately 1700 in 2016 to about 2100 in 2019 (p < 0.05).

**Conclusions:**

Twitter engagement metrics at the Academic Surgical Congress 2015–2019 has fluctuated, while impressions significantly increased through the years indicating the consistent dissemination of conference content.

## Highlights

• Cross sectional review of the Academic Surgical Congress's online reach and engagement through the use of Twitter hashtags #ASC 2015–2019.
• Twitter engagement metrics at the Academic Surgical Congress 2015–2019 has fluctuated, while impressions significantly increased through the years.
• Using Twitter to maximize the dissemination of scientific surgical research and evidence-based practices is highly beneficial to medical professionals.

## 1 Background

The use of Twitter at medical conferences has revolutionized the way healthcare professionals advance their education. Instant dissemination of new ideas and online interactions through the use of Twitter hashtags has greatly magnified the reach of medical conferences materials. Twitter is uniquely suited to improve communication and spread of ideas at conferences because of its simple, free access and interactive format. Users publish tweets with a 280-character limit that can include identifying hashtags, that other users can “like,” retweet and respond to. In addition to the text, users can include pictures, videos and links in their tweets.

Twitter provides the unique opportunity for physicians to build an online brand or personality. This has improved opportunities for connections and even mentorship with medical trainees or other professionals [1]. The same study also found that Twitter puts inexperienced attendees on the same platform as presenters who are often experts on the topics that they are presenting. The use of physician influencers to increase the utilization of the conference hashtag by having them tweet meeting content and engage participants who may not be in attendance has also proven effective [[2], [3], [4]]. Additionally, Twitter has been shown in the literature to provide several benefits towards the dissemination of academic medicine [1,5]. A study by Djuricich coined the term “evidence-based tweeting” which allows physicians to tweet newly discovered findings and provide the relevant literature in subsequent tweets [5]. This has furthered reach into medical conferences where attendees are now equipped with tools to quickly spread this novel information.

Many studies have shown that Twitter improved medical conferences online participation from year to year by documenting increasing numbers of tweets, users and impressions [[2], [6], [7], [8], [9], [10], [11], [12], [13]]. One study found that after promoting use of a hashtag at the conference, their organizational profile showed a 20% increase in followers over the next week [14]. Understanding the impact of the use of Twitter on surgical conferences is invaluable to advance education in the field of surgery. Despite this, data regarding the use of Twitter at surgical conferences is limited.

Using conference hashtags to increase a conference's online presence benefits not only those in attendance but those following the conference proceedings remotely. Through a targeted emphasis on the online impact of medical conferences, we can help ensure those who are unable to attend conferences due to work or time constraints are still able to learn from the novel ideas presented.

In this study, we aim to investigate the scope of the Academic Surgical Congress's (ASCs) online reach and engagement through the use of Twitter hashtags #ASC from 2015 to 2019. We measure this by analyzing the number of impressions (reachability metrics measured in millions) and engagement (tweets & retweets metrics measured in thousands). The ASC is a combined meeting of the two leading associations for academic surgery – The Association for Academic Surgery (AAS) and The Society of University Surgeons (SUS). As a prominent international surgical conference, which is greatly attended, the potential for impact is more significant than many other conferences that have been previously analyzed. Additionally, there have been no studies investigating the reach of the ASCs Twitter use over the period of multiple years. We hypothesize that increased Twitter impressions and engagements will result in wide dissemination of knowledge/conference material and increase the conference attendance rate.

## 2 Methods

A cross sectional review of Twitter data at the ASC from 2015 to 2019. Symplur Signals software (Upland,CA), designed specifically for hashtags related to healthcare topics was used to review and analyze studied Twitter metrics. We analyzed the following conference hashtags: #ASC2015, #ASC2016, #ASC2017, #ASC2018 and #ASC2019. From this analysis, we obtained information on the number of engagements, impressions, and users who participated by using the conference hashtags. Only publicly available twitter data metrics were utilized for analysis. This study was reviewed by our Institutional Review Board (IRB) and the Western Institutional Review Board (WIRB) and deemed exempt.

Data obtained from Symplur was analyzed to obtain a more thorough understanding of the breadth of Twitter use at the ASC from 2015 to 2019. The term “impression” is defined as the number of people who only viewed a tweet containing the conference hashtag, but did not engage. In contrast, “engagement” is defined as action on conference content through user tweets containing the conference hashtag or “likes”, retweets or comments on a Tweet containing the conference hashtag. “Twitter influencers”, who are accounts of either organizations or individuals, are able to gather a significant amount of impressions and tweet engagement due to their large number of followers. Therefore, we investigated the role of twitter influencers on hashtag engagement. These influencers are defined through Symplur as having high conference hashtag utilization. This study was reported in line with the Strengthening the Reporting of Cohort Studies in Surgery (STROCSS) 2019 guidelines [15].

## 3 Results

Impressions continued to significantly grow during the time period of 2015–2019, from 13,100 to 39,900 to 63,100 to 65,900 to peak at 71,800 impressions in 2019, respectively. From 2015 to 2017 there was a consistent increase in engagement from 11,400 to 27,700 to 32,100, respectively. However, from 2017 to 2019, there was a significant decline in engagement from 32,100 to 26,100. Data regarding tweets, retweets, users, impressions and Top 3 Links tweeted are summarized in Table 1.

**Table 1:** Twitter impressions and engagement, users and top shared links/articles for #ASC 2015–2019.

Year	Tweets (in thousands)	Retweets (in thousands)	Users (in thousands)	Impressions (in millions)	Top 3 articles
2015	7.7 k	3.9 k	1.5 k	13.1 *m*	1. Twitter 101: How to set up a professional Twitter account 2. Essential surgery: key messages from Disease Control priorities 3. ASC Searchable Abstracts
2016	16.9 k	10.8 k	3.8 k	39.9 *m*	1. National Cluster-Randomized Trial of Duty-Hour Flexibility in Surgical Training 2. Surgical Resident Duty Hour Rules- Weighing the New Evidence 3. Extending the Length of Surgical Trainees Shifts does not affect Surgical Patients Safety
2017	19.2 k	12.9 k	4.6 k	63.1 *m*	1. AAS Presidential Address 2. AAS Statement on Diversity, Scientific Development and International Fellowship 3. Is there still a Glass Ceiling for women in Academic Surgery?
2018	16.8 k	11.7 k	3.7 k	65.9 *m*	1. Medscape 2. 16th Annual Academic Surgical Congress 2. Lessons learned from the 2018 Academic Surgical Congress 3. Behind the Knife podcast
2019	15.2 k	10.9 k	3.3 k	71.8 *m*	1. Global burden of postoperative death 2. National Evaluation of Gender Discrimination and Sexual Harassment in US General Surgery Residency Programs 3. Annual Student Conference: Thinking Three Moves Ahead: Setting your Sights on Advanced and Consultant practice

The term “users” refers to Twitter accounts that participated in meeting conversation by tweeting the conference hashtag. Users grew from 1500 in 2015 and 3800 in 2016, to peak at 4600 in 2017 before dipping back to 3700 in 2018 and 3300 in 2019.

Symplur signals provides data on the Most Tweeted Links containing the meeting hashtag. The Most Tweeted Links represent articles that were presented at the conference, however there were a significant amount of outdated links that have since been deleted as our study refers back to posts dating as far back as 2015.

The Top 10 Twitter influencers by year were primarily occupied by the Association for Academic Surgery (@Academic Surgery, AAS), followed by the Society of University Surgeons (@UnivSurgeon, SUS). Other contributing organizations included the Association of Women Surgeons (@WomenSurgeons, AWS). The remaining influencers were individual physicians at varying academic levels and from a myriad of surgical specialties as summarized in Table 2.

**Table 2:** Top ten influencers for #ASC 2015–2019.

	2015	2016	2017	2018	2019
1	Association for Academic Surgery	Association for Academic Surgery	Association for Academic Surgery	Association for Academic Surgery	Association for Academic Surgery
2	Acute Care Surgeon A	General Surgeon A	Surgical Oncologist B	Society of University Surgeons	Society of University Surgeons
3	Pediatric Surgeon A	Society of University Surgeons	General Surgeon A	General Surgeon A	Cardiothoracic Surgeon B
4	Thoracic Surgeon A	Trauma Surgeon A	Trauma Surgeon A	Women Surgeons, Organization	Trauma Surgeon A
5	Burn and Critical Care Surgeon A	General Surgeon B	Society of University Surgeons	Thoracic Surgeon A	Association of Women Surgeons
6	Surgical Oncologist A	Acute Care Surgeon A	General Surgeon D	Surgical Oncologist A	Surgical Oncology/Endocrine Surgeon A
7	Trauma Surgeon A	Health Services Researcher A	Association of Women Surgeons	Endocrine Surgeon A	Trauma Surgeon C
8	Society of University Surgeons	Trauma and Critical Care Surgeon B	Trauma Surgeon A	Trauma Surgeon A	Vascular Surgeon A
9	General Surgeon A	Thoracic Surgeon A	Thoracic Surgeon A	Acute Care Surgeon A	General Surgeon E
10	Surgical Oncologist B	General Surgeon C	General Surgeon B	Burn and Critical Care Surgeon A	Breast and General Surgeon A

**Fig. 1:**
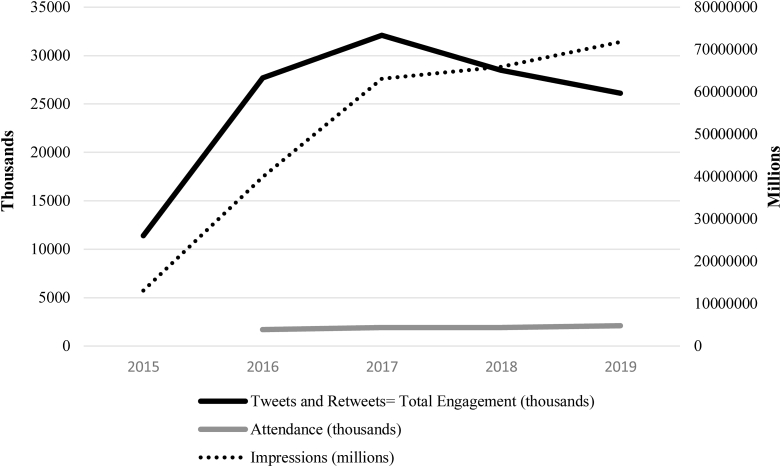
Total Online Engagement, Impressions and Attendance at the ASC 2015-2019. Impressions, Total Engagement (tweets & retweets combined), and attendance at the ASC 2015–2019. Total engagement and impressions grew significantly 2015–2017. Impressions continued its growth while total engagement declined 2017–2019. Attendance mildly increased from 2016 to 2019.

Fig. 1 represents the relationship between total engagement (tweets and retweets combined), impressions, and attendance rate. Due to their nature, engagement signifies a more involved degree of interaction while impression denotes more passivity. This comparison is highly indicative of online involvement as compared to conference attendance. Total engagement increased from 11,400 in 2015 to 27,700 in 2016 to 32,100 in 2017 and then declined to 28,500 in 2018 and 26,100 in 2019. However, impressions steadily increased from 2015 to 2019, from 13.1 million to 39.9 million to 63.1 million to 65.9 million to 71.8 million, respectively. Attendance also increased steadily throughout the years, from 1700 in 2016 to 1900 in 2017 and 2018 to 2100 in 2019. Attendance data in regards to the year 2015 was not publicly available. Engagement did not reflect the increased attendance but instead peaked in 2017 and dropped from 2017 to 2019. It is important to note that Twitter engagement were not related to the same year's attendance, but to the following year. While we cannot definitively conclude that the use of Twitter increases meeting attendance rates, there is a defined relationship between increasing number of impressions and conference attendance rates.

Understanding where the majority of the conference tweets originate is crucial to the discussion on engagement. In this study, engagement is defined as the number of “likes”, tweets, retweets, or comments; however, these numbers may not reflect continuous conversation or involvement as active as it may appear. For example, in 2017 when engagement was the highest, 71% of users only used the conference hashtag once, which does not indicate active participation or conversation. These numbers remained largely the same, even in 2019, when only 13.5% of users tweeted more than 5 times.

## 4 Discussion

Through an analysis of hashtag data usage and interaction for the ASC from the years of 2015–2019, we aimed to investigate relationships between online impact and attendance levels. Our study found that though engagement peaked in 2017 and declined subsequently in 2018–2019, impressions continued to increase. This exhibits that while Twitter interaction may have decreased in more recent years, users were still viewing conference content. One phenomenon to explain the increasing impression but decreased engagement is social media overload and fatigue [16]. Social media overload and fatigue are a result of overstimulation of the individual from social media sites, friends and followers, and too much time spent online maintaining these connections. While the overstimulation continues to be represented by the increasing number of impressions, there will be a decrease in the amount of engagements as this requires a more active form of connection. The number of users peaked in 2017, however, 2016 saw the highest percentage of users tweeting more than 5 times, indicating active participation in online conversation related to the conference, at 18% of users. Attendance increased steadily through the years, regardless of the location of the conferences. Our results demonstrate a possible relationship between Twitter usage and increasing attendance through study period.

Engagement significantly decreased from the years of 2017–2019 by nearly 6000. There are several possible explanations for this phenomenon. First, an insufficient level of twitter participation or interest in utilization of conference hashtags in more recent years may have influenced engagement numbers during this time. As we discovered, attendance did not grow between 2017 and 2018 and minimally grew in 2019, which may also affect Twitter engagements. In addition to the social media overload and fatigue, overall Twitter usage patterns over the same time interval. According to Twitter data AI experts, Twitter usage and membership began to stagnate in 2018, prior to which the social media site was gaining users and engagement [17,18]. Twitter engagement may have stagnated also in part due to policies and ways of advertisement for hashtag promotion. Displaying the hashtag on other conference materials such as name tags, agendas, and programming and even on lecture slides also has the potential to increase awareness and boost Twitter use. It is also presumable that attendees simply did not remember to include the hashtag when tweeting about conference materials. This may be addressed by widespread encouragement and promotion of hashtag inclusion into tweets. Additionally, using physician influencers to boost Twitter use and start online conversations may also increase participation [19,20]. Finally, content published from meetings may not have been published in a visually attractive or easily understandable format which could have led to lower interaction. Including links to videos, infographics and pictures in addition to commentary can increase a tweet's appeal.

As Twitter has the potential to include participants nationally and globally, it has a particularly unique role in enhancing engagement in an international conference such as the ASC. In a study of the Canadian Geriatrics Conference, tweets were separated by content such as conference sessions, networking, resource sharing, and conference promotion. This study also found that 60% of Twitter participants were not Canadian, indicating an international reach [21]. Up to 26% of Twitter participation in the Irish Society of Urology's National Meeting were by virtual followers not attending the conference, indicating the expansive reach that internet communication allows [22]. Similarly, the American Urology Society surveyed urologists, 33% of whom stated that they have participated in a Urology conference remotely through Twitter [23]. At the 2016 conference on the Science of Dissemination and Implementation in Health, it was found that of the 2639 tweets related to the conference, 22 locations outside of the U.S. were represented [24]. In 2014, Cochran et al. published on the impact of Twitter at the 2013 ASC meeting. They discovered that there were 434 tweets containing that years' hashtag, and of the 37 users that were identifiable, only 51% were in actual attendance at the ASC 2013 meeting [25]. This suggests the impact that hashtags and twitter engagement can have on stirring discussion and conversation with those unable to attend the conference thereby widening its impact as shown in other medical-society meetings. While this has been the major work on social media usage at the ASC conference until now, our analysis of multiple years allowed us to track change in engagement and impressions over time and elucidate further details on engagement.

A study of 14 Anesthesiology conferences showed no relationship between meeting attendance, registration, total tweets, impressions and retweets. However, a positive correlation was demonstrated between the number of Twitter users participating using the conference hashtags and the Twitter metrics of tweets, retweets, impressions and comments [17]. Additionally, a study by Rabarison et al. found that the use of hashtags helped to promote conversation between healthcare experts and their audience, by providing an organized space for discourse [26]. Patients or other non-physician users who interact and with conference hashtags have the ability to further propagate conversation and information [27]. The usage of hashtags and efforts to track engagement allows for discernment of which topics or presentations may be the most impactful or important as they may represent large foci of the conversation [28].

We recommend that scientific medical meetings use Twitter to increase their online participation of those attending and those following along remotely in order to increase knowledge dissemination. Ensuring that the hashtag is advertised on Twitter and on the association's website can boost hashtag use before and during the conference [29]. Disseminating information via social media also allows the conference to become more streamlined. Physical attendees can still be updated on a topic of their interest while attending a different session at that same conference, preventing a gap in knowledge from not physically being present during that specific session [30].

This study has several limitations. Data regarding conference attendance in 2015 was not available. In addition, information may have been tweeted regarding the conference where the hashtag was not used. Conversely, hashtags may have been utilized in tweets not containing relevant conference information. Symplur Signals is not an exhaustive software, and certain information could not be obtained from our analysis, such as the occupation of each individual user who interacted with meeting content. Therefore we were unable to ascertain which tweets are from medical professionals versus the general public. Filters created by Symplur signals were utilized to provide data only on Tweets published in English and in the North American time zone. Importantly, Twitter engagement relates to the previous years’ attendance. Finally, there may be a proportion of meeting participants and attendees who do not utilize social media or Twitter for academic discussions.

The recent COVID-19 pandemic has clearly shown that such sudden unanticipated disruptions that lead to health and safety concerns result in the cancellation of upcoming medical meetings, conferences and training courses. This has led to renewed interest for consideration of developing a virtual web-based platform to minimize the impact of not being able to attend these educational symposia in person. Twitter and other social media platforms may serve as an important alternative to continuing to disseminate the educational component of these meetings. We theorize that these internet-based services will continue to grow in importance and may provide a resilient solution to any future global health crises and/or inclement climate issues, while potentially minimizing missed educational opportunities. They will continue to provide an easily accessible network to allowing meeting content to be available for remote audiences. We also suspect that engagement will increase during these times as the transition to remote conferences will encourage active participation by conference attendees. Developing these innovative alternative options is crucial to continue the spread of medical research knowledge and improved clinical care despite impediments.

Twitter serves as a free of cost online forum to increase the interactions and engagement between surgeons, facilitate the spread of new research ideas and advance the scientific learning process. As social media is constantly growing in the evolution of our digital society, we anticipate that the role of Twitter will continue to increase in conferences in the future.

## 5 Conclusion

Twitter engagement metrics and number of users at the ASC has fluctuated through the years, however the consistent increase in numbers of Twitter impressions show conference content can still be widely disseminated. Twitter hashtag usage may have contributed to the increased in attendance rates throughout the years. Using Twitter to maximize the dissemination of scientific surgical research and evidence-based practices and guidelines is highly beneficial to all medical professionals, particularly surgeon scientists, leading to not only improving their scientific knowledge and research skills but also ultimately translating into improved patient care.

## Ethical approval

This study was conducted in compliance with ethical standards, reviewed by our institutional review board and the Western Institutional Review Board and deemed exempt.

## Sources of funding

None.

## Author contribution

AE, KS, MM– Conception of study, acquisition of data, analysis and interpretation of data

KS, AE, AS, EM, DB, MM – drafting of manuscript, critical revision of manuscript.

KS, EM, AS, AE, DB, MM – Approval of the final version for submission.

## Research registration number

researchregistry5961.

## Guarantor

Mark McKenney.

## Provenance and peer review

Not commissioned, externally peer reviewed.

## Declaration of competing interest

Authors declare no competing interests.
